# Loss of Skeletal Muscle Mass During Neoadjuvant Chemoradiotherapy Predicts Postoperative Mortality in Esophageal Cancer Surgery

**DOI:** 10.1245/s10434-015-4558-4

**Published:** 2015-04-17

**Authors:** Kostan W. Reisinger, Joanna W. A. M. Bosmans, Martine Uittenbogaart, Abdulaziz Alsoumali, Martijn Poeze, Meindert N. Sosef, Joep P. M. Derikx

**Affiliations:** Department of Surgery, Maastricht University Medical Center, Maastricht, The Netherlands; NUTRIM School for Nutrition, Toxicology and Metabolism, Maastricht University, Maastricht, The Netherlands; Department of Surgery, Atrium Medical Center, Heerlen, The Netherlands

## Abstract

**Background:**

Esophageal surgery is associated with complications and mortality. It is highly important to develop tools predicting unfavorable postoperative outcome. Esophageal cancer and neoadjuvant chemoradiotherapy (CRT) induce skeletal muscle wasting, which leads to diminished physiologic reserves. The purpose of this study was to investigate whether the degree of muscle mass lost during neoadjuvant CRT predicts postoperative mortality.

**Methods:**

A total of 123 consecutive patients undergoing surgery for esophageal malignancy in the period 2008–2012 were included, of whom 114 received neoadjuvant CRT. Skeletal muscle mass was measured on routinely performed CT scans by assessing L3 muscle index (according to the Prado method) before and after neoadjuvant CRT, and the amount of muscle mass lost during neoadjuvant CRT (muscle loss index) was calculated. It was investigated whether this amount was associated with postoperative 30-day or in-hospital mortality and morbidity.

**Results:**

In the complete cohort, no significant association between loss of muscle mass and mortality was found. However, skeletal muscle mass was significantly lower in patients with stage III–IV tumors compared with stage I–II tumors, prior to neoadjuvant CRT. In the stage III–IV subgroup, the amount of muscle mass lost during neoadjuvant CRT was predictive of postoperative mortality: −13.5 % (standard deviation 6.2 %) in patients who died postoperatively compared with −5.0 % (standard deviation 8.3 %) in surviving patients, *p* = 0.02.

**Conclusions:**

Measurement of muscle mass loss during neoadjuvant chemoradiotherapy may provide a readily available and inexpensive assessment to identify patients at risk for developing unfavorable postoperative outcome after resection of esophageal malignancies, especially in patients with stage III–IV tumors.

Complications after esophageal resection present a serious problem: 26–41 % of patients develop major adverse events; the majority of which are cardiopulmonary and infectious complications or anastomotic leakage. Mortality rates are 4–10 %.^1^ Therefore, it is imperative to develop tools that predict unfavorable postoperative outcome adequately. The assessment of sarcopenia may indicate whether complications are likely to arise. Sarcopenia is defined as a syndrome characterized by progressive and generalized loss of skeletal muscle mass and strength, which increases the risk of adverse health-related outcomes, such as impaired physical ability, decreased quality of life, and mortality.^2^,^3^ We showed before that in colorectal surgery, sarcopenia as an important element of functional compromise is associated with postoperative mortality.^4^ Neoadjuvant chemoradiotherapy (CRT) induces significant sarcopenia in esophageal cancer patients.^5^ Moreover, esophageal cancer can further deteriorate skeletal muscle mass by causing cachexia. Sarcopenia is associated with complications following surgery.^6^,^7^ Furthermore, sarcopenic obesity, defined as high body mass index and low muscle tissue on CT scan, is hypothesized to be a predictor of unfavorable oncologic and surgical outcomes.^8^,^9^ It is known that both sarcopenia and obesity are associated with chronic inflammation, which could explain the high incidence of postoperative complications in sarcopenic obese patients.^10^,^11^ In addition, these patients are often not seen as “at risk,” because normal body mass index due to high fat mass camouflages sarcopenia.

A readily available instrument to quantify skeletal muscle mass is CT-based muscle area measurement at the level of the lumbar three vertebral landmark, which can be accomplished by using open-source software.^12^,^13^ Although low preoperative muscle mass measured on CT scans could not be associated with mortality and length of hospital stay in patients undergoing esophageal surgery, the amount of muscle mass loss induced by neoadjuvant CRT may be a more dynamic predictor of postoperative outcome.^5^ Increased muscle loss before surgery presumably reflects high metabolic stress due to malnutrition, cachexia, tumor load, or inflammation, negatively affecting the metabolic response to major gastrointestinal surgery. In this study, it was hypothesized that the amount of muscle mass loss during neoadjuvant CRT predicts postoperative mortality after esophageal resection. The primary purpose of the study was to measure whether the amount of muscle mass lost during neoadjuvant CRT in patients undergoing esophageal resection was associated with postoperative mortality. The secondary goal of the study was to measure whether sarcopenic obesity and fat loss over the course of neoadjuvant CRT, using a pre- and post-CRT time point, was associated with postoperative mortality.

## Methods

### Patients

All patients who underwent resection of esophageal cancer in a single teaching hospital from January 2008 until January 2012 were enrolled in a prospectively maintained, digital database. Data included characteristics of the primary tumor and oncologic staging, specifications of surgical treatment, chemotherapy, radiotherapy, and postoperative complications. All patients who received neoadjuvant CRT were included in this study. Neoadjuvant CRT consisted of three cycles of cisplatin/5-fluorouracil (CF) or five cycles of paclitaxel/carboplatin (PC), all with concurrent radiotherapy, or consisted of three cycles of epirubicin/cisplatin/capecitabine (ECC), also with concurrent radiotherapy in some patients. A routine total body PET/CT scan before neoadjuvant CRT (preCRT) and after neoadjuvant CRT (postCRT) was performed, as standard care for all patients diagnosed with esophageal cancer. After a multidisciplinary meeting, which took place weekly, patients were admitted to the neoadjuvant CRT protocol, generally meaning that treatment started within 2 weeks after PET/CT. The second PET/CT was performed directly after neoadjuvant CRT, following the same protocol in all patients. The average time between the first and second PET/CT was 111 days [standard deviation (SD), 13 days]. The variation was mostly due to neoadjuvant CRT taking longer because of side-effects. Patients were closely monitored by a dietician from the moment of diagnosis until hospital discharge after surgery, and malnutrition was screened for using the Short Nutritional Assessment Questionnaire (SNAQ). Adequate oral intake was verbally encouraged, and if nutritional depletion was present, patients received protein-rich and lipid-rich drinks. A gastric or jejunal feeding tube was placed in patients with esophageal stenosis. Furthermore, physical activity was stimulated by counseling or by active preconditioning by an expert physiotherapist. The study was conducted with approval from local medical ethical committees and according to the revised version of the Declaration of Helsinki (October 2008, Seoul). The principles of good clinical practice (GCP) were followed during this study.

### Postoperative Mortality

The primary clinical outcome of the study was postoperative mortality. This was defined as mortality within 30 days or within hospital admission.

### CT-Based Muscle and Fat Measurements

CT-based measurements were performed using Osirix^®^ Version 3.3 (32-bit; http://www.osirix-viewer.com) by a researcher who was blinded from outcome. The cross-sectional skeletal muscle surface (cm^2^) as measure of sarcopenia was measured at the level of the third lumbar vertebra (L3) on two consecutive transversal slices on which both vertebral spines were visible.^13^ The “Grow Region (2D/3D Segmentation)” tool in the menu of the program facilitated to select automatically all skeletal muscle mass in one slice. In the same manner, L3 total adipose tissue was composed of all adipose tissue on the L3 level, including subcutaneous adipose tissue, visceral adipose tissue, and intramuscular adipose tissue. Visceral and subcutaneous fat were measured after manual adjustment to ensure no excessive tissue was included in the measurements. The average surface (cm^2^) of the two consecutive slices was used for the analyses. The distinction between different tissues was based on Hounsfield units (HU) with a threshold range of −29 to +150 HU used for skeletal muscle and a range of −190 to −30 HU for fat tissue.^7^,^12^ Muscles measured were: psoas, quadratus lumborum, paraspinal, transverse abdominal, external oblique, internal oblique, and rectus abdominis muscles. Hand-adjustment of the selected areas was performed if necessary and the values of muscle or fat area were automatically calculated by Osirix^®^^13^

### Sarcopenia and Muscle Loss Index

The L3 muscle area surfaces were normalized for patient height to calculate the L3 muscle index and expressed in cm^2^/m^2^. Patient height and weight were measured by a nurse at admission to the hospital at the time of diagnosis. The cutoff values used for sarcopenia were 52.4 cm^2^/m^2^ for men and 38.5 cm^2^/m^2^ for women, based on the method of Prado et al.^12^ Previous data showed high interobserver agreement with kappa of sarcopenia assessment by CT image analysis using Osirix^®^ of 0.87 (95 % confidence interval 0.82–0.93) and Bland–Altman analysis produced 95 % limits of agreement: −5.9 to 8.7 %.^14^

The muscle loss index (MLI), as a measure of muscle mass loss during chemoradiotherapy, was calculated as follows: (postCRT L3 index—preCRT L3 index)/preCRT L3 index × 100 %.

### Visceral Obesity and Sarcopenic Obesity

Sex-specific cutoff values for visceral obesity were 163.8 cm^2^ for men and 80.1 cm^2^ for women.^15^ For sarcopenic obesity, two separate definitions were used. The first definition was the combination of sarcopenia and visceral obesity (sarcopenic visceral obesity); the second definition was the combination of sarcopenia and obesity based on BMI > 30 kg/m^2^ (sarcopenic obesity). Visceral obesity and sarcopenic obesity were calculated at both time points (preCRT and postCRT), using height and weight values recorded at the time of diagnosis.

### Statistical Analysis

Normality was tested using Kolmogorov–Smirnov. Frequencies are presented as absolute numbers and percentages. Continuous data are presented as mean and standard deviation (SD). Differences between groups were analyzed with the Pearson *χ*^2^ test for dichotomous parameters. Paired *t* test was used for between group comparison of preCRT and postCRT continuous data. Independent samples *t* test was used for between group comparison of continuous data on a single time point (preCRT or postCRT) and for between-group comparison of MLI and fat loss. Receiver operating characteristic (ROC) curves were used to calculate accuracy of the relative L3 index decrease to predict postoperative mortality. The ideal cutoff value for predicting mortality was defined as the cutoff value with maximum sum of sensitivity and specificity. Overall diagnostic accuracy was represented by the area under the curve (AUC). Two-tailed *p* values <0.05 were considered significant. All statistical analyses were performed using SPSS^®^ 20.0 (SPSS Inc, Chicago, IL).

## Results

### Patients and L3 Index During Chemoradiotherapy

**Fig. 1:**
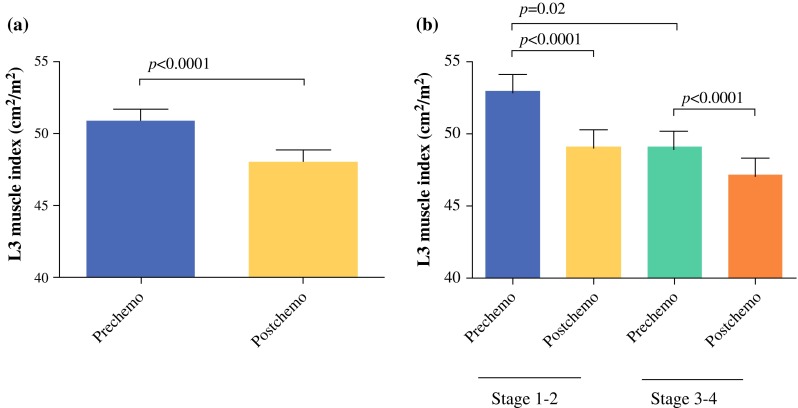
L3 muscle index before and after neoadjuvant CRT in patients undergoing esophageal surgery. **a** Patients with both a preCRT and a postCRT CT scan (*n* = 96). **b** By tumor stage: stage III*–*IV (*n* = 52) versus I–II (*n* = 44)

**Diagram 1:**
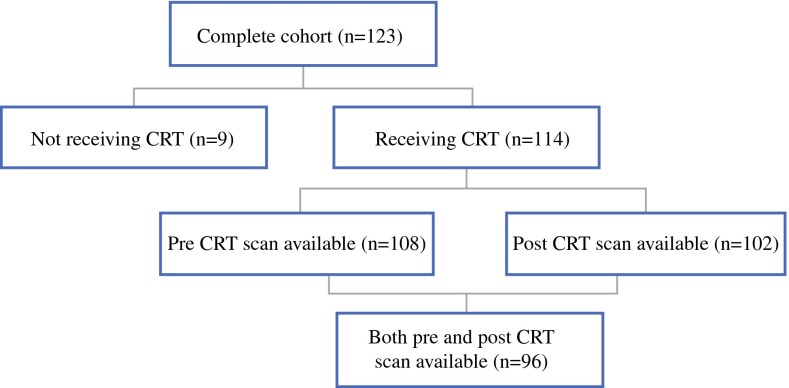
Proportion of patients having preCRT and postCRT scans

A total of 123 patients were included, of whom 101 (82 %) were males; 114 patients received neoadjuvant CRT. Both a preCRT and postCRT CT scan were available in 96 patients. Patient, tumor, and operation characteristics are presented in Table 1. An explanatory flowchart of patients, including those receiving CRT and availability of CT scans, is presented in Diagram 1. Sarcopenia was present in 60 of 108 patients (56 %) before CRT and in 74 of 111 patients (67 %) after CRT. Concomitantly, the mean L3-index decreased significantly during CRT, which was measured in patients with both a preCRT and a postCRT CT scan [50.9 (SD, 8.5) cm^2^/m^2^ to 48.4 (8.5) cm^2^/m^2^, *p* < 0.001; Fig. 1a]. In male subjects, the L3-index decreased from 53.4 (7.8) to 49.5 (7.9) cm^2^/m^2^, *p* < 0.001, and in female subjects the L3-index decreased from 42.7 (5.4) to 39.7 (4.4) cm^2^/m^2^, *p* = 0.02. Mortality within 30 days or within hospital admission was 11 of 123 (9 %) in the total cohort and 6 of 62 (10 %) in patients with stage III-IV tumors. Of note, only stage IV patients who responded well to CRT and were classified as stage III after CRT were eligible for surgery. Of patients with both a preCRT and postCRT CT scan, mortality rates were 9 of 96 (9 %) and 5 of 52 (10 %), respectively. Causes of mortality are listed in Table 2.

**Table 1:** Patient, tumor, and operation characteristics

	Complete cohort (*n* = 123)		Patients with preCRT and postCRT CT scans (*n* = 96)	
	Number of patients	Mean (SD)	Number of patients	Mean (SD)
Sex				
Male	101 (82.1 %)		80 (83.3 %)	
Female	22 (17.9 %)		16 (16.7 %)	
Age (year)		63 (10)		63 (9.2)
>70	32 (26 %)		23 (24 %)	
BMI (kg/m^2^)		24.7 (4.2)		24.7 (4.3)
<18.5	12 (9.7 %)		10 (10.4 %)	
18.5–24.9	54 (43.9 %)		42 (43.8 %)	
25.0–29.9	44 (35.8 %)		32 (33.3 %)	
≥30.0	13 (10.6 %)		12 (12.5 %)	
Length of hospital stay (days)		26 (33)		26 (35)
ASA				
I	30 (25.4 %)		28 (29.2 %)	
II	52 (44.1 %)		44 (45.8 %)	
III	36 (30.5 %)		24 (25.0 %)	
Stage				
I–II	61 (49.6 %)		44 (45.8 %)	
III–IV	62 (50.4 %)		52 (54.2 %)	
Time between preCRT CT and postCRT CT (days)		111 (17)		111 (17)
Time between postCRT CT and operation (days)		33 (19)		32 (19)
Type of chemotherapy				
CF	99 (86.8 %)		84 (87.5 %)	
ECC	6 (5.2 %)		3 (3.1 %)	
PC	9 (7.9 %)		9 (9.4 %)	
Tumor type				
Adenocarcinoma	100 (81.3 %)		82 (85.4 %)	
Squamous cell carcinoma	23 (18.7 %)		14 (14.6 %)	
Tumor location				
Proximal esophageal	1 (0.9 %)		16 (16.7 %)	
Mid esophageal	20 (17.9 %)		53 (55.3 %)	
Distal esophageal	57 (48.7 %)		21 (21.9 %)	
Junctional	29 (24.8 %)		6 (6.3 %)	
Gastric	10 (8.5 %)		44 (45.8 %)	
Type of surgery				
Transthoracic	58 (47.2 %)		52 (54.2 %)	
Transhiatal	65 (52.8 %)			664 (1,085)
Blood loss (mL)		664 (970)		

*ASA* American Society of Anesthesiologists, *CRT* chemoradiotherapy, *CF* cisplatin/5-fluorouracil, *ECC* epirubicin/cisplatin/capecitabine, *PC* paclitaxel/carboplatin, *BMI* body mass index, calculated at time of diagnosis

**Table 2:** Causes of mortality

	Number of patients
Anastomotic leakage	5
Pneumonia and respiratory failure	3
Intestinal ischemia	1
Cardiac ischemia	1
Postoperative pulmonary bleeding	1

### Influence of Tumor Characteristics on the L3 Index

The mean L3 index was significantly lower before CRT began in patients with advanced (stage III–IV) tumors compared with localized tumors [49.1 (8.4) cm^2^/m^2^ and 52.9 (8.4) cm^2^/m^2^, *p* = 0.02; Fig. 1b]. (Male subjects: 54.3 (7.8) compared with 51.5 (7.6) cm^2^/m^2^, *p* = 0.02; female subjects: 44.8 (7.5) compared with 40.0 (3.3) cm^2^/m^2^, *p* = 0.03), respectively.) In both stage III–IV and stage I–II tumors, the L3 index decreased significantly during CRT. L3 indices were not different between patients with adenocarcinoma and patients with squamous cell carcinoma, and there was no difference between the amount of sarcopenic patients between carcinoma types.

### L3 Index Decrease as a Predictor of Mortality

**Fig. 2:**
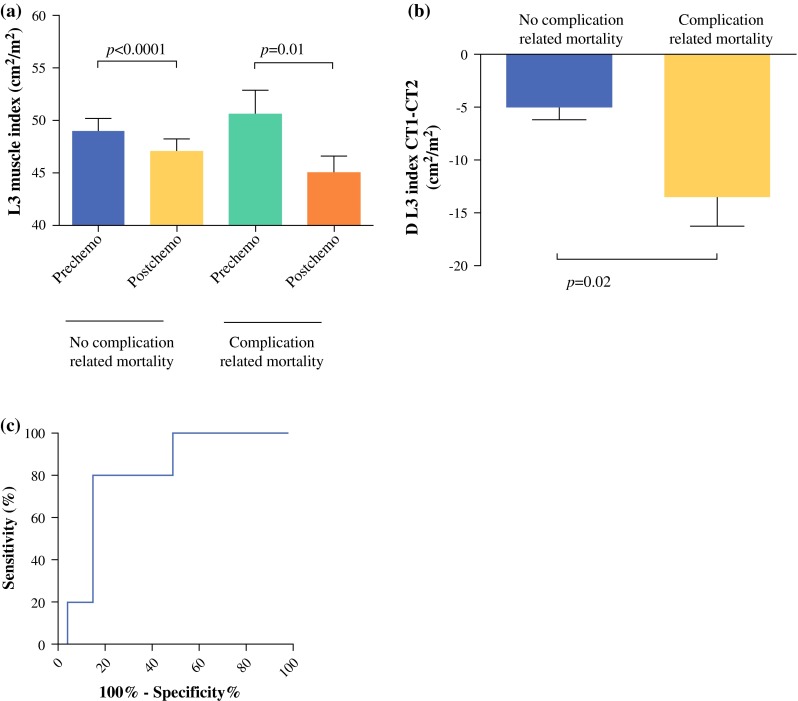
Loss of muscle mass measured as decrease of L3 muscle index in patients with advanced (stage III–IV) tumors. **a** L3 indices before and after CRT in surviving patients (*n* = 47) and patients who die from postoperative complications (*n* = 5). **b** Relative L3 index decrease in surviving patients and patients who die from postoperative complications. **c** ROC curve of relative L3 index decrease to predict postoperative mortality

The MLI was not predictive for postoperative mortality directly related to complications in the total study population; −8.6 % (7.6 %) in patients who died postoperatively compared with −7.0 % (8.0 %; *p* = 0.57) in surviving patients. Because L3 indices differed significantly between stage III–IV and stage I–II disease, these groups were analyzed separately. In patients with stage I-II tumors, MLI was not different between patients with and those without postoperative mortality, respectively: −2.5 % (3.6 %) and −9.4 % (7.1 %), *p* = 0.07. In patients with stage III–IV tumors, patients who died postoperatively showed significantly higher decrease in MLI [−13.5 % (6.2 %)] compared with surviving patients [−5.0 % (8.3 %), *p* = 0.02; Figs. 2a, b]. An analysis for female patients could not be done, because there were no stage III–IV female patients with postoperative mortality in this cohort. Analysis of only male patients yielded comparable results.

MLI was not associated with incidence of specific complications (anastomotic leakage, abscess, empyema, sepsis, postoperative ileus, surgical site infections, pneumonia, and pneumothorax) or with length of hospital stay. To determine diagnostic accuracy of the MLI to detect postoperative mortality in patients with stage III–IV disease, ROC curve analysis was done (Fig. 2c). An optimal cutoff point of −13.2 % was found with sensitivity 80 %, specificity 85 %, positive likelihood ratio 5.33 [95 % confidence interval (CI), 2.5–11.0], and negative likelihood ratio 0.24 (95 % CI 0.04–1.4; *p* = 0.03). The area under the ROC curve was 0.80 (95 % CI 0.64–0.96).

### Sarcopenic Obesity as a Predictor of Postoperative Mortality

At the preCRT time point, 16 patients were defined as having sarcopenic visceral obesity (sarcopenia and visceral obesity) and two patients were defined as having sarcopenic obesity (sarcopenia and high BMI; Table 3). At the postCRT time point, these were 20 and 2 patients, respectively. Sarcopenic visceral obesity was not associated with postoperative mortality (preCRT, *p* = 0.79; postCRT, *p* = 0.46). Furthermore, sarcopenic obesity was not associated with mortality (preCRT, *p* = 0.66; postCRT, *p* = 0.66). When patients with stage I–II tumors and those with stage III–IV tumors were analyzed separately, comparable results were found.

**Table 3:** Body composition measures

	Number of patients	Mean (SD)
L3 index (cm^2^/m^2^)		
preCRT		50.9 (8.5)
postCRT		48.4 (8.5)
Visceral fat (cm^2^)		
preCRT		159.0 (98.5)
postCRT		148.0 (89.5)
Subcutaneous fat (cm^2^)		
preCRT		159.8 (79.5)
postCRT		153.0 (80.5)
Sarcopenic obesity		
preCRT	2 (2 %)	
postCRT	2 (2 %)	
Sarcopenic visceral obesity		
preCRT	16 (17 %)	
postCRT	20 (21 %)	

Height and weight values used for all calculations were recorded at time of diagnosis

### Fat Loss as a Predictor of Postoperative Mortality

In contrast to loss of muscle mass, total fat loss was not associated with postoperative mortality in the complete study cohort, nor when stage I–II disease and stage III-IV disease were analyzed separately (stage I–II: −9.1 % (20.0 %) in patients who died postoperatively compared with −4.8 % (24.2 %) in surviving patients, *p* = 0.74; stage III–IV: −21.8 % (23.4 %) compared with −3.0 % (38.2 %), respectively, *p* = 0.29). Comparable results were found for subcutaneous fat and visceral fat.

## Discussion

This study confirms the loss of muscle mass during neoadjuvant CRT in patients who undergo resection of esophageal malignancies. This is the first study that shows that loss of muscle mass, as measured on routinely obtained CT scans, is associated with postoperative mortality in patients with advanced tumors. This may provide a readily available and inexpensive assessment to identify patients at risk for developing unfavorable postoperative outcome. Intriguingly, none of the anthropometric parameters at a single time point were significantly associated with postoperative outcome, but rather the dynamic loss during neoadjuvant CRT. It is therefore important to calculate the MLI, as a difference between preCRT and postCRT. Although hypothesized, sarcopenic obesity was not associated with postoperative mortality in this study. The MLI seemed to be of more importance than the combination of sarcopenia and high BMI. No association could be found between loss of fat mass of any type and postoperative mortality. The mortality rate in this study is comparable to reported rates in recent literature, where percentages vary from 5 to 10 %.^16^,^17^

The application of patient-tailored approaches is upcoming in oncologic health care.^18^ In this context, measurements of the physical ability to recover adequately from therapeutic hits, such as surgery, is vital, particularly in the tumor-bearing patient in whom metabolic and nutritional resources are generally depleted. Considering the striking impact of metastasized disease on physiological depletion, patients with stage III and IV tumors are more prone to loss of reserves, manifesting as cachexia, which includes loss of muscle mass. The current data reflect this effect by lower L3 muscle index in patients with stage III–IV disease. This loss in muscle tissue is the result of both increased muscle protein degradation and reduced muscle synthesis. Tumors, especially in more advanced stages, may increase host’s resting energy expenditure (REE). Ravasco et al. found an elevated REE in patients with stage III–IV colorectal tumors.^19^ Furthermore, Bachmann et al. showed that pancreatic cancer patients with cachexia had a higher rate of more progressed tumor stages and a worse nutritional status.^20^ Selection of patients with increased risk for poor postoperative outcome within this vulnerable group therefore is important. Delaying the moment of surgery and with application of prehabilitation programs to regain physical resources or preventive postoperative ICU admissions may offer advantages for patients who are identified as high risk for poor outcome. During this study, patients who were diagnosed with and treated for esophageal cancer received standard care. Regarding physical activity, this means that they have received physical therapy during their hospital admission, but no additional physical intervention was provided.

The inability of a hospital to let patients with severe complications survive is known as failure to rescue (FTR). Instead of higher complication rates, higher FTR rates are the main determinant of postoperative mortality.^21^ FTR variability between hospitals is largely contributed to hospital-related factors, such as the available level of ICU care.^22^ On the other hand, variability of patient characteristics influences FTR rates as well.^23^ The amount of muscle mass lost during neoadjuvant CRT could be used as a predictor of FTR rates in future studies.

Our data are in line with several studies. Deans and colleagues showed that weight loss, among others, was independently predictive of death after esophageal resections.^24^ This study focused on long-term mortality (disease prognosis); therefore, weight loss may actually be a reflection of tumor progression rather than functional depletion. In their recent study, Awad et al. showed a decrease in muscle mass during CRT as measured on the L3 level on CT scans, although a correlation with adverse postoperative outcome could not be found.^5^ Patients with advanced tumors were not analyzed separately from those with local tumors, as in the current study.

As with every observational study, the current data should be taken with caution. Although the studied cohort was relatively large, numbers of complication-related deaths are small. Therefore, the observed effect should be validated in a larger, preferably multicenter or nationwide cohort. Second, sarcopenia rates were relatively high in this study using the predefined cutoff values that remain subject to debate. It is desirable to define cutoff values based on different cancer type populations. Finally, muscle and fat loss are only one aspect of functional depletion. Other parameters, such as loss of muscle function and strength, were not measured in this study. This is unfortunate, because a recent report showed that function tests may assess muscle quality, making their relationship with prognosis potentially better than that of muscle mass.^25^

It should be addressed whether attempts to attenuate loss of muscle mass during neoadjuvant CRT are effective and whether they result in decreased FTR rates. In the studied population, efforts to counteract exhaustion of physiologic reserves by nutritional support were already done, indicating that nutrition alone is not the key to success. Several systematic reviews and meta-analyses have concluded that physical exercise may improve physical functioning and overall quality of life in cancer patients.^26^,^27^ Strength training is the most effective exercise to slow down the rate of loss of muscle mass and maintaining and improving muscle strength. The combination of physical exercise with essential amino acid ingestion elicits the greatest anabolic response.^28^,^29^ Because physical exercise is only effective in terms of improving muscle strength when performed 2–3 days a week, the feasibility of this approach in combination with the physical and mental side-effects of neoadjuvant CRT should be determined.^30^ Finally, eicosapentaenoic acid (EPA) can preserve lean body mass after esophageal surgery and therefore may be an interesting option to preserve muscle mass during CRT.^31^
